# Levels of physical activity and sedentary time among 10- to 12-year-old boys and girls across 5 European countries using accelerometers: an observational study within the ENERGY-project

**DOI:** 10.1186/1479-5868-9-34

**Published:** 2012-03-31

**Authors:** Maïté Verloigne, Wendy Van Lippevelde, Lea Maes, Mine Yıldırım, Mai Chinapaw, Yannis Manios, Odysseas Androutsos, Éva Kovács, Bettina Bringolf-Isler, Johannes Brug, Ilse De Bourdeaudhuij

**Affiliations:** 1 Department of Movement and Sport Sciences, Ghent University, Ghent, Belgium; 2 Department of Public Health, Ghent University, Ghent, Belgium; 3 Department of Public and Occupational Health and the EMGO Institute for Health & Care Research, VU University Medical Center, Amsterdam, the Netherlands; 4 Department of Nutrition and Dietetics, Harokopio University, Athens, Greece; 5 Department of Paediatrics, University of Pécs, Pécs, Hungary; 6 Department of Epidemiology and Public Health, Swiss TPH, Basel, Switzerland; 7 University of Basel, Basel, Switzerland; 8 Department of Epidemiology and Biostatistics and the EMGO Institute for Health & Care Research, VU University Medical Center, Amsterdam, the Netherlands

## Abstract

**Background:**

The study aim was to objectively assess levels of sedentary time, light, moderate and vigorous physical activity (PA) among 10-12 year olds across five European countries and to examine differences in sedentary time and PA according to gender and country.

**Methods:**

686 children (mean age = 11.6 ± 0.8 years, 53% girls, mean BMI = 19.0 ± 3.4 kg/m^2^) from Belgium, Greece, Hungary, the Netherlands and Switzerland wore Actigraph accelerometers and had at least 2 weekdays with minimum 10 h-wearing time and 1 weekend day with minimum 8 h-wearing time. Data were analyzed using multivariate analyses of covariance.

**Results:**

Girls spent significantly more time sedentary (500 minutes/day) than boys (474 minutes/day) and significantly less time in light (267 minutes/day) and moderate-to-vigorous PA (32 minutes/day) than boys (284 minutes/day; 43 minutes/day respectively; p < 0.001). 4.6% of the girls and 16.8% of the boys met moderate-to-vigorous PA recommendations of at least 60 minutes/day. Greek boys were more sedentary (510 minutes/day; all at p < 0.05) than other boys. Dutch girls were less sedentary (457 minutes/day; all at p < 0.05) than other girls. Swiss girls displayed more moderate-to-vigorous PA (43 minutes/day; at p < 0.05) than other girls.

**Conclusions:**

Large proportions of children across different European countries did not meet PA recommendations and spent a lot of time sedentary. Mean time spent in moderate-to-vigorous PA was significantly lower than the recommended 60 minutes. Obesity prevention programmes focusing on both decreasing sedentary time and increasing light, moderate and vigorous PA are needed for European children, particularly girls.

## Introduction

Physical activity (PA) is important for physical and mental health, and is acknowledged as an important target in obesity prevention [1-3]. According to this evidence, it has been recommended for children to participate in at least 60 minutes per day moderate-to-vigorous intensity PA (MVPA) [4-6]. For a long time, being sufficiently physically active was considered the opposite of having a sedentary lifestyle. However, high levels of MVPA are not necessarily equal to low levels of sedentary time, given that children meeting MVPA recommendations can still be sedentary for many hours per day [7,8]. A previous study revealed that some youngsters engaged in many hours of sport participation per week but, at the same time, watched TV or played on the computer for more than four hours per day [7]. It is therefore evident that PA and sedentary time should be regarded as two different behaviours. Moreover, recent studies showed that both behaviours independently contribute to the development of chronic diseases and overweight and obesity [9-12]. Although there are no clear-cut recommendations in place for sedentary time, reducing sitting time and screen viewing behaviours is recommended. Obesity prevention programmes should therefore develop strategies to minimize the time spent sedentary, besides promoting PA.

True levels of PA and sedentary time are difficult to assess in population-based studies. Until recently, such assessments relied on self-reports with low validity and reliability [13]. To objectively assess accurate levels of both PA and sedentary time, accelerometers are nowadays available and more frequently used [14]. Accelerometers are considered valid and reliable instruments and feasible to use in large-scale observational and intervention studies [14,15]. Accelerometry enables distinguishing between different levels of PA (light, moderate, vigorous) and between light PA (LPA) and sedentary time [16]. Nevertheless, accelerometry in cross-country observational epidemiological research is still rare. One previous study, as part of the European Youth Heart Study, has compared MVPA levels of children across four European countries using accelerometers [17]. However, this study did not specifically focus on the time spent sedentary or on LPA while it is of major importance to assess those behaviours as well. To date, no other large cross-European study has brought both children's sedentary time as well as different intensities of PA into focus.

The objective of the current study is therefore to objectively assess levels of sedentary time, LPA and MVPA in 10- to 12-year-old children across five European countries using accelerometers. Differences in sedentary time, LPA and MVPA according to gender and country were examined as well. We specifically focused on 10- to 12-year-old children since the transition from childhood to adolescence represents a critical period for declining PA levels and increasing sedentary time [18,19].

## Methods

### Participants

Participants in the present study were part of a larger European cross-sectional survey within the framework of the ENERGY-project ("EuropeaN Energy balance Research to prevent excessive weight Gain among Youth"-project), which has been thoroughly described elsewhere [20]. The cross-sectional survey aimed at gathering up-to-date information on the prevalence of overweight and obesity and on energy balance-related behaviours and their correlates across seven European countries. Originally, the seven European countries were Belgium, Greece, Hungary, the Netherlands, Norway, Slovenia and Spain. In the course of the ENERGY-project, Switzerland agreed to participate in the cross-sectional survey as well, following the same standard protocol. Per country, three cities were selected with a different degree of urbanization (low, middle and high tertile). Schools were randomly selected in the three cities to reach a representative sample of 1000 children, aged between 10 and 12 years old. Additionally, five countries -Belgium, Greece, Hungary, the Netherlands and Switzerland- collected accelerometer data in a subsample of preferably 200 children to gather objective information on PA and sedentary time. The number of selected schools for accelerometry was proportional with the number of schools in the selected cities for the cross-sectional survey. Data collection occurred on schooldays between March and September 2010 (data collection from March-July took place before the summer holiday whereas data collection from August-September took place after the summer holiday). Each research team complied with the ethical procedure of their country. The protocol of the ENERGY cross-sectional study has been described by van Stralen et al. [21].

### Measurement

#### Instrumentation

Sedentary time and PA were assessed using three models of Actigraph (Pensacola, FL) accelerometers: the uniaxial accelerometer GT1M and the triaxial accelerometers GT3X (dimensions: 3.8 cm × 3.7 cm × 1.8 cm) and Actitrainer (dimensions: 8.6 cm × 3.3 cm × 1.5 cm). The proportions of these three models of accelerometers used in each country are provided in Table 1. Since two triaxial and one uniaxial accelerometer model was used, we only made use of the vertical axis output for the present study. The Actitrainer and the GT3X have identical triaxial accelerometers. Furthermore, a recent study confirmed that the vertical axis output for the GT3X is similar as for the GT1M [22]. The three accelerometers are lightweight devices -weighing respectively 27, 27 and 51 grams- and are worn on the right hip secured by an elastic waist belt. In general, Actigraph accelerometers have shown adequate reproducibility, validity and feasibility in children and adolescents [23].

**Table 1:** Proportions of the Actigraph accelerometers models used per country

	GT1M	GT3X	Actitrainer
Belgium (n = 109)	0%	96%	4%
Greece (n = 142)	0%	0%	100%
Hungary (n = 140)	67%	0%	33%
The Netherlands (n = 104)	0%	0%	100%
Switzerland (n = 192)	37%	63%	0%
**Total sample (n = 687)**	**33%**	**43%**	**24%**

#### Measurement protocol

Researchers responsible for the accelerometer data collection were trained to work according to a standardized accelerometer protocol. Accelerometers were initialized using ActiLife software [24]. A 15-sec recording epoch was selected in order to capture short duration PA of high intensity [25,26]. Children wore the accelerometer for six consecutive days, including two weekend days. In the Swiss subsample, they wore the accelerometer for seven consecutive days (two weekend days included). Children wore the accelerometer during all waking hours, but removed it during water-based activities. At the end of the data collection, all accelerometer data were transferred to VU University Medical Center in the Netherlands for data processing, i.e. data reduction and analysis. The software Meterplus 4.2 was used to screen and clean the accelerometer data files of the six days measurement [27]. Non-wearing time was calculated as periods of more than 20 minutes of consecutive zero counts. Children were included in the study if they had at least 2 weekdays with minimum 10 h-wearing time and 1 weekend day with minimum 8 h-wearing time. The average counts per 15 seconds provided information on the overall activity level. Minutes per day (average of all valid days) of sedentary time, LPA, moderate PA (MPA) and vigorous PA (VPA) were estimated using the cut-points from Treuth et al. [28]: < 100 counts per minute (cpm) equals sedentary time, 101-2999 cpm equals LPA, 3000-5199 cpm equals MPA and > 5200 cpm equals VPA. Reilly et al. [29] suggested that the most appropriate cut-point for MPA lies in the range 3000-3600 cpm. A recent study of Trost et al. [30] stated that the cut-point of 100 cpm for sedentary time exhibited good to excellent classification accuracy. The protocol of accelerometry in the ENERGY-project is thoroughly described elsewhere [31].

### Anthropometric measurements

Body weight was measured to the nearest 0.1 kg with a calibrated electronic scale (SECA, 861) with the children wearing light weight clothing and no shoes. Body height was measured to the nearest 0.1 cm with a Seca Leicester Portable stadiometer. The body mass index (BMI) in kilogram per square meter (kg/m^2^) was calculated on the basis of height (m) and weight (kg) measures.

### Statistical analyses

All statistical analyses were performed using SPSS 15.0 (SPSS Inc, Chicago, IL). Time spent in MVPA was dichotomized into 0 (< 60 minutes MVPA per day) and 1 (≥ 60 minutes MVPA per day). Means, standard deviations and/or proportions were calculated for age, BMI, wearing time, counts per 15 seconds, sedentary time, LPA, MPA, VPA, MVPA and percentage of children meeting PA recommendations. A multivariate analysis of covariance (MANCOVA) was used to determine whether counts per 15 seconds, sedentary time, LPA, MPA, VPA, MVPA varied across genders and across countries. Four covariates were included: (a) age, (b) month of wearing the accelerometer (since Switzerland has started somewhat later with the accelerometer data collection), (c) accelerometer model (since different accelerometer models were used across countries) and (d) language spoken at home (native vs. non-native) as an indicator of ethnicity. When significant main effects were found, least significance difference (LSD) pairwise comparisons were made. A statistical significance level of 0.05 was used in all analyses.

## Results

In total, 1082 children have worn the accelerometer across five countries from which 766 children provided valid data. Among the omitted children, 52 did not have sufficient weekdays, 141 did not have sufficient weekend days and 115 failed in achieving sufficient week- and weekend days. Eight accelerometers gave an error while downloading data. Of those 766 children, 80 children did not have BMI, age and/or ethnicity data, bringing the final number to 686 children (365 girls and 321 boys).

### Gender differences

Table 2 shows means of age, BMI, wearing time, counts per 15 seconds, sedentary time, LPA, MPA, VPA and MVPA for boys and girls separately (indicated in bold). After adjusting for age, girls spent significantly more time sedentary and less time in all PA intensities than boys (all at p < 0.001).

**Table 2:** Age, BMI, wearing time, counts per 15 seconds, sedentary time and the different PA intensities + percentages of children meeting PA guidelines of 60 minutes MVPA per day (for boys and girls separately per country)

Gender	Country	Age (years) (mean ± SD)	BMI (kg/m^2^) (mean ± SD)	Wearing time (total days) (mean ± SD)	Counts per 15 sec (mean ± SE)	Sedentary time^1^ (mean ± SE)	LPA^1^ (mean ± SE)	MPA^1^ (mean ± SE)	VPA^1^ (mean ± SE)	MVPA^1^ (mean ± SE)	Meeting PA guidelines (%)
Girls (n = 365)	Belgium (n = 59)	11.3 ± 0.7	17.5 ± 2.2	5.9 ± 0.9	121 ± 6^b, e^	511 ± 11^d^	263 ± 8	17 ± 2^c, e^	6 ± 1	23 ± 2^c, e^	1.7
	Greece (n = 79)	11.3 ± 0.6	20.3 ± 3.6	5.4 ± 0.9	106 ± 5^a, c, d, e^	526 ± 9^c, d^	269 ± 7	20 ± 2^c, e^	5 ± 1^c, e^	25 ± 2^c, e^	0.0
	Hungary (n = 68)	12.2 ± 0.6	19.7 ± 3.1	5.2 ± 0.9	139 ± 6^b, d^	487 ± 9^b, d^	260 ± 7	29 ± 2^a, b, d, e^	8 ± 1^b, d^	37 ± 2^a, b, d, e^	1.5
	The Netherlands (n = 47)	11.8 ± 0.5	18.8 ± 3.1	5.2 ± 1.3	123 ± 5^b, c, e^	457 ± 9^a, b, c, e^	278 ± 7	21 ± 1^c, e^	4 ± 1^c, e^	26 ± 2^c, e^	2.1
	Switzerland (n = 112)	11.3 ± 0.9	17.6 ± 2.7	6.5 ± 1.0	145 ± 5^a, b, d^	498 ± 9^d^	270 ± 6	35 ± 1^a, b, c, d^	8 ± 1^b, d^	43 ± 2^a, b, c, d^	12.5
	**TOTAL GIRLS**	**11.5 ± 0.8**	**18.7 ± 3.2**	**5.8 ± 1.1**	**128 ± 2^f^**	**500 ± 3^f^**	**267 ± 3^f^**	**26 ± 1^f^**	**6 ± 0^f^**	**32 ± 1^f^**	**4.6**
Boys (n = 321)	Belgium (n = 50)	11.5 ± 0.7	18.5 ± 2.9	5.8 ± 1.0	159 ± 8^b^	478 ± 12^b^	290 ± 2	31 ± 2^e^	10 ± 1^b^	42 ± 3	14.0
	Greece (n = 63)	11.3 ± 0.6	21.2 ± 4.2	5.4 ± 0.8	140 ± 7^a, d, e^	510 ± 11^a, c, d, e^	282 ± 2	34 ± 2	7 ± 1^a^	41 ± 3	9.5
	Hungary (n = 72)	12.2 ± 0.7	20.0 ± 3.5	5.4 ± 0.9	145 ± 6^d^	475 ± 10^b, d^	272 ± 2	34 ± 2^e^	7 ± 1	41 ± 3^e^	13.9
	The Netherlands (n = 57)	11.7 ± 0.6	18.7 ± 3.4	5.5 ± 1.3	162 ± 6^b, c^	447 ± 9^b, c^	290 ± 2	32 ± 2^e^	8 ± 1	40 ± 2^e^	15.8
	Switzerland (n = 79)	11.5 ± 1.0	17.9 ± 3.1	6.6 ± 1.3	164 ± 7^b^	467 ± 11^b^	282 ± 2	40 ± 2^a, c, d^	10 ± 1	50 ± 3^c, d^	27.8
	**TOTAL BOYS**	**11.7 ± 0.8**	**19.2 ± 3.6**	**5.8 ± 1.2**	**155 ± 2^g^**	**474 ± 4^g^**	**284 ± 3^g^**	**35 ± 1^g^**	**9 ± 0^g^**	**43 ± 1^g^**	**16.8**

^1 ^minutes per day; *SD *standard deviations; *SE *standard errors; *LPA *light physical activity; *MPA *moderate physical activity; *VPA *vigorous physical activity; *MVPA *moderate-to-vigorous physical activity; ^a ^significantly different from Belgium; ^b ^significantly different from Greece; ^c ^significantly different from Hungary; ^d ^significantly different from the Netherlands; ^e ^significantly different from Switzerland; ^f ^significantly different from boys; ^g ^significantly different from girls.

### Country differences

**Figure 1:**
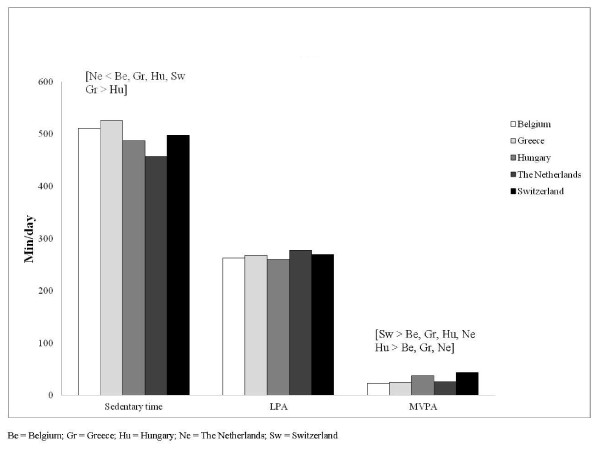
**Mean levels of sedentary time, LPA and MVPA among girls**.

**Figure 2:**
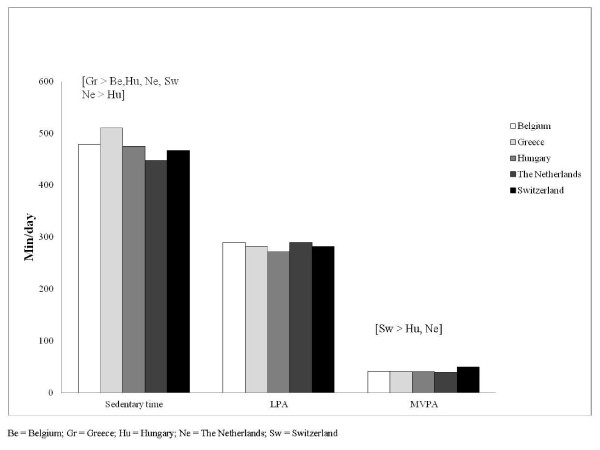
**Mean levels of sedentary time, LPA and MVPA among boys**.

Table 2 shows the mean values for every country separately. After adjusting for age, accelerometer model, month of wearing and language spoken at home, Greek boys were more sedentary (510 minutes per day) than all other boys (all at p < 0.05). Greek girls spent the most time sedentary (526 minutes per day), although they did not significantly differ from Belgian (511 minutes per day) and Swiss girls (498 minutes per day). Dutch girls were less sedentary than all other girls with an average of 457 minutes per day (all at p < 0.05). Swiss girls spent more time in MVPA than all other girls with 43 minutes per day (all at p < 0.05). Swiss boys spent the most minutes in MVPA (50 minutes per day), although they did not significantly differ from Belgian (42 minutes per day) and Greek boys (41 minutes per day). No significant differences were found in LPA between the five countries. Figure 1 (girls) and Figure 2 (boys) give an visual overview of the mean levels of sedentary time, LPA and MVPA for every country separately.

### MVPA recommendations

4.6% of the girls in the current study met the recommendation of at least 60 minutes MVPA per day, compared to 16.8% of the boys (see Table 2). None of the Greek girls met the MVPA recommendations. For Belgian, Hungarian and Dutch girls, the percentage of girls meeting MVPA recommendations ranged from 1.5 to 2.1%, whereas this was 12.5% in Swiss girls. In boys, approximately one fourth of the Swiss boys met MVPA recommendations (27.8%). The lowest percentage of boys meeting MVPA recommendations was found in Greece: 9.5%.

## Discussion

The current study provides for the first time up-to-date information on objectively assessed sedentary time and different PA intensities among representative samples of 10- to 12-year-old children across five countries in Europe. The results show that very few boys and girls across Europe met the recommendations of 60 minutes MVPA per day and that they spent approximately eight hours per day being sedentary. Boys did somewhat better than girls, and rather large differences between countries were observed. The results strongly confirm that obesity prevention programs focusing on promoting PA and reducing sedentary time in this age group are needed and such efforts should be further intensified. Although evidence-based recommendations for maximum levels of sedentary time do not exist, eight hours per day certainly appears to be a bit too much sitting, especially for young people. Thus, interventions focusing particularly on reducing sitting time, next to promoting PA, should be considered. As it may not be feasible to replace a lot of time spent sedentary by MVPA, it could be advisable to promote LPA as an alternative to sedentary pursuits [32]. For adults, replacing sedentary activities by PA of light intensity, such as standing and walking around, appeared to be a practicable strategy [33]. For children, there is the added evidence that LPA contributes to total daily energy expenditure, resulting in beneficial associations with blood glucose and overweight [16,34]. A recent study of Janz et al. [35] showed that LPA may have a beneficial effect against excess adiposity in older children. Nevertheless, future research should further investigate whether replacing sedentary activities by LPA could have an impact on children's health. Furthermore, since the predominant activity at school is sitting in class [34], more innovative intervention strategies may be considered such as breaking up sedentary time between lessons and changing the school or class environment [36].

Regarding the time spent in MVPA, the mean level among European children was far below recommendations. Only 4.6% of the European girls and 16.8% of the European boys reached the recommended 60 minutes MVPA per day. These percentages are alarming and imply an urgent need to develop effective PA promotion programs. Riddoch et al. [17] found that almost all 9-year-old children across four European countries fulfilled MVPA recommendations. However, different cut-points were used in that study (> 906 cpm equals moderate activity), classifying activities with a lower intensity value already as 'moderate' intensity. More severe cut-points were used in the present study [28] which makes it impossible to compare results between the two cross-European studies.

Comparable to previous studies [37-39], the current study established gender differences in both sedentary time and PA across all countries. Girls spent more time being sedentary and less time in all PA intensities than boys. These gender differences in the transition from childhood to adolescence are the precursors of the larger gender differences in adolescence [37]. Intervention programmes to promote PA and to reduce sedentary time should therefore merit special attention to girls. For example, the NEAT girls intervention has recently developed a specific programme to promote PA among girls with lifestyle physical activities and appealing school sport sessions (e.g. yoga, Pilates, skipping choreography) [40]. However, we are not aware of intervention programmes to reduce sedentary time among girls specifically. Future research should further investigate the specific reasons of the differences in PA and sedentary time among girls and boys.

Furthermore, the current study disclosed some cross-European differences in the levels of sedentary time and PA, possibly due to political and environmental differences. In general, Greek children spent more time sedentary in comparison to the other children and Swiss children spent more minutes in MVPA in comparison to the other children. Especially for Swiss children, there is a large difference in MVPA with the other European countries. A possible explanation for differences between countries is the different organization of PA and sports (e.g. financial issues and spending priorities in the field of sport). The Swiss national sports policy for example considers 'health' as the first priority with more physically active people as the main objective [41]. There is also a national sports programme 'Youth and Sport' which offers optional physical education lessons after school [42]. However, not all differences are situated on country-level, since PA facilities and opportunities differ between schools as well. Physical education can be considered to have a major influence on children's PA time [43], e.g. Hungarian and Swiss schools provide three hours of physical education per week, whereas Belgian, Greek and Dutch schools provide two hours per week. In brief, schools play a major role in PA promotion. To our knowledge, there are no policy measures in the European countries with regard to sedentary time. However, political institutions should be made aware of the importance of the time children spent sedentary and should definitely pay attention to this topic in the future [44]. Despite some country differences, obesity prevention programmes focusing on sedentary time and PA are clearly needed in all European countries. Time spent sedentary was namely high in every country and the mean amount of minutes MVPA was below the recommended 60 minutes for almost all children.

The most important strengths of the current study were the specific focus on sedentary time and LPA, the relatively large sample of children across several European countries, the use of accelerometry to objectively assess different levels of PA and sedentary time and the use of a standard protocol for data collection and data processing. However, when using accelerometers, there are some limitations as well: accelerometers do not measure arm movement or swimming activities, do not distinguish between lying, sitting and standing still and underestimate the intensity of cycling and some other activities. A second limitation of the current study is the relatively large drop-out of accelerometer-data due to insufficient valid days. It is possible that the children who dropped out were less physically active and not interested in wearing an accelerometer or conversely, were more active and experienced the accelerometer as an inconvenience during exercise. A final possible limitation is the use of three different models of Actigraph accelerometers (GT1M, GT3X and Actitrainer). Since two triaxial and one uniaxial accelerometer model was used, we only made use of the vertical axis output for the present study. The Actitrainer and the GT3X have identical triaxial accelerometers and a recent study showed that the vertical axis output for the GT3X is similar as for the GT1M [22]. However, it has also been posited that the reduced sensitivity in low-count ranges in the GT1M may make it somewhat less suited for monitoring sedentary time and LPA [45].

## Conclusion

In conclusion, the present study shows low levels of PA and high sedentary time in schoolchildren in five countries across Europe. The study emphasizes the need for continuous and further intensified efforts to promote PA and for interventions focusing specifically on reducing sedentary time. Future research should now focus on the specific time period when children are most likely to be sedentary and least likely to be physically active (weekday vs. weekend day, during school vs. after school) to optimize the intervention programmes as much as possible.

## Competing interests

The authors declare that they have no competing interests.

## Authors' contributions

JB developed the concept and design of the ENERGY-project. MV has conducted the analyses and drafted the paper. IDB has significantly contributed to the final manuscript by introducing new discussion points. All other co-authors have been involved in the coordination and/or implementation of the ENERGY accelerometer study. All authors read and approved the final manuscript.
